# Inpatient COVID-19 mortality has reduced over time: Results from an observational cohort

**DOI:** 10.1371/journal.pone.0261142

**Published:** 2022-01-13

**Authors:** Katie Bechman, Mark Yates, Kirsty Mann, Deepak Nagra, Laura-Jane Smith, Andy I. Rutherford, Amit Patel, Jimstan Periselneris, David Walder, Richard J. B. Dobson, Zeljko Kraljevic, James H. T. Teo, William Bernal, Richard Barker, James B. Galloway, Sam Norton

**Affiliations:** 1 Centre for Rheumatic Diseases, King’s College London, London, United Kingdom; 2 King’s College Hospital NHS Foundation Trust, London, United Kingdom; 3 Department of Biostatistics and Health Informatics, Institute of Psychiatry, Psychology and Neuroscience, King’s College London, London, United Kingdom; 4 NIHR Biomedical Research Centre at South London and Maudsley NHS Foundation Trust and King’s College London, London, United Kingdom; 5 Health Data Research UK London, University College London, London, United Kingdom; 6 School of Immunology and Microbial Sciences, King’s College London, London, United Kingdom; 7 Psychology Department, King’s College London, London, United Kingdom; Stanford University School of Medicine, UNITED STATES

## Abstract

**Background:**

The Covid-19 pandemic in the United Kingdom has seen two waves; the first starting in March 2020 and the second in late October 2020. It is not known whether outcomes for those admitted with severe Covid were different in the first and second waves.

**Methods:**

The study population comprised all patients admitted to a 1,500-bed London Hospital Trust between March 2020 and March 2021, who tested positive for Covid-19 by PCR within 3-days of admissions. Primary outcome was death within 28-days of admission. Socio-demographics (age, sex, ethnicity), hypertension, diabetes, obesity, baseline physiological observations, CRP, neutrophil, chest x-ray abnormality, remdesivir and dexamethasone were incorporated as co-variates. Proportional subhazards models compared mortality risk between wave 1 and wave 2. Cox-proportional hazard model with propensity score adjustment were used to compare mortality in patients prescribed remdesivir and dexamethasone.

**Results:**

There were 3,949 COVID-19 admissions, 3,195 hospital discharges and 733 deaths. There were notable differences in age, ethnicity, comorbidities, and admission disease severity between wave 1 and wave 2. Twenty-eight-day mortality was higher during wave 1 (26.1% versus 13.1%). Mortality risk adjusted for co-variates was significantly lower in wave 2 compared to wave 1 [adjSHR 0.49 (0.37, 0.65) p<0.001]. Analysis of treatment impact did not show statistically different effects of remdesivir [HR 0.84 (95%CI 0.65, 1.08), p = 0.17] or dexamethasone [HR 0.97 (95%CI 0.70, 1.35) p = 0.87].

**Conclusion:**

There has been substantial improvements in COVID-19 mortality in the second wave, even accounting for demographics, comorbidity, and disease severity. Neither dexamethasone nor remdesivir appeared to be key explanatory factors, although there may be unmeasured confounding present.

## Background

The United Kingdom (UK) has seen two discrete waves of COVID-19. The first commenced in March 2020 and the second in late October 2020. Admissions to hospital in the second wave exceeded those during the first. At the peak of the first wave (12th April 2020), there were 21,684 patients in hospital with COVID-19 across the UK compared to 39,220 at the peak of the second wave (18th January 2021) [1]. The number of patients in critical care was higher in the first wave, accounting for 15% of total hospital admissions compared to 10% at the peak of wave 2 [1].

Many studies examined patient characteristics and predictors of mortality during the first wave. The OpenSafely group identified increasing age, deprivation, comorbidity, and ethnicity as predictors of COVID-19 mortality using UK primary care records linked to hospital episode statistics [2]. Other work, including from our centre, has incorporated patient observations, laboratory blood parameters and radiographic findings to model risk of critical care admission or death in patients admitted with COVID-19 [3, 4].

As we have learnt more about SARS-CoV-2, new treatment options have become available. Dexamethasone and remdesivir have been licensed (June and July 2020 respectively) after showing efficacy in randomised clinical trials [5, 6]. Clinicians are more familiar with the natural history of the disease and guidelines have been published regarding admission criteria, oxygen therapy and anticoagulation [7–10]. During the second wave a new variant of SARS-CoV-2 (variant of concern (VOC) 202012/01) appeared to accelerate transmission. This led to speculation as to whether this variant had a higher mortality [11].

We have used detailed clinical data from a 1,500-bed London Hospital Trust to address the following questions: Were the characteristics of those admitted with COVID-19 in the second wave different to the first? Was the mortality rate in the second wave different to the first? Were any differences in survival attributable to dexamethasone or remdesivir?

## Methods

### Study oversight

This project operated under London South East Research Ethics Committee (reference 18/LO/2048) approval granted to the King’s Electronic Records Research Interface (KERRI); specific work on COVID-19 research was reviewed with expert patient input on a virtual committee with Caldicott Guardian oversight. The need for consent was waived by ethical approval as no identifiable data was evaluated or individual persons data presented.

### Data sources

This study represents an observational cohort of two London hospitals (King’s College Hospital, Camberwell and Princess Royal University Hospital, Bromley) reflecting a South London catchment population of approximately 1.2 million. Data were captured through routine care in a single electronic health record instance (Sunrise Clinical Manager, Allscripts). We included all patients aged 16 and over, admitted as an emergency with COVID-19 infection between 1st March 2020 and 26th February 2021. Patients were excluded if they were admitted for an elective procedure and had an incidental positive COVID-19 swab or if their first positive swab was more than three days after hospital admission, suggesting possible nosocomial infection.

Self-identified ethnicity was coded as White, Black (including Black African and Black Caribbean), Asian, or other (including Mixed). Comorbidities were extracted via natural language processing (NLP) of text records as used in previous published studies [12, 13] and additionally supplemented with manual validations of medical records [14]. Chest X-ray abnormalities used an NLP-approach of radiologist reports identifying any features of consolidation, opacities, lobar shadowing, pleural effusion and ground-glass changes. Clinical parameters including age, gender, and physiological observations [15], C-reactive protein (CRP) and neutrophil count on admission, were also captured from the EHR.

### Study endpoints

The primary outcome was death within 28 days of hospital admission. If a patient was readmitted within seven days of discharge, the admission durations were combined and considered a single episode. If readmission occurred more than seven days after initial discharge, the second episode was excluded from analysis. Follow up was censored up until 6^th^ May 2021 for patients still admitted. Patients discharged within seven days of this date only contributed data up until their date of discharge, as ascertainment of deaths outside hospital could not be guaranteed in this time window due to time delays in reporting.

### Statistical methods

Hospital admissions over the study duration were graphically presented and three distinct time periods were identified: wave 1, inter-wave period, wave 2. Periods representing the start of a “wave” of hospital admission were defined by five or more admissions over two consecutive days, whilst the end of the wave was defined by fewer than five admissions on two consecutive days. Characteristics of patients admitted in each wave were tabulated and tested for statistically significant imbalance using Chi-square, Mann–Whitney or t-tests, as appropriate.

Changes in the risk of death for patients admitted with COVID-19 over time were analysed using time-to-event data competing risks regression models, estimated using the Fine and Gray method [16]. The event of interest was death during admission whilst the competing risk was discharge from hospital. A competing risks model indicates the probability of the outcome, accounting for the probability that patients may also succumb to a separate event. This ensures the cumulative incidence function is correctly estimated.

Individuals were considered ‘at risk’ from the date of admission for 28 days, or until date of death or discharge, whichever came first. Proportional sub-hazards models were used to compare the mortality risk across the time periods. The first peak of admissions (wave 1) was used as the reference group. Multivariate adjustment was made for the following covariates: age, sex, ethnicity, comorbidity (obesity, diabetes and hypertension), and admission physiological observations score, CRP, neutrophils and chest radiographs score, as well as dexamethasone and remdesivir use during their inpatient stay. Admission CRP levels and neutrophils counts were chosen a priori. These laboratory biomarkers are strong predictors of COVID-19 related critical care admission or death and have been used to identify patients at risk of deterioration. Compared to other serum measures including d-dimer and LDL, nearly all patients had a CRP and neutrophil result from the day of admission. Lymphocyte counts were not included as these have shown a non-linear association, with both low and high counts associating with COVID-19 disease severity, Chest radiographs were assessed using an adapted radiographic assessment of lung oedema (RALE) score for COVID-19, as introduced by Wong et al. [17]. The severity score attributes a number between 0–4 to each lung depending on extent of consolidation or ground glass opacities (0 = no involvement, 1 = <25%, 2 = 25–49%, 3 = 50–75%, 4 = >75% involvement). Values for each lung were summed to produce a final score ranging from 0–8.

To examine the impact of new therapeutic options for COVID-19, a Cox proportional hazard model without competing risks (as the hazard ratio was the parameter of interest, which would not be biased by competing events) was used to compare mortality in patients prescribed remdesivir or dexamethasone. Individuals were considered ‘at risk’ from the date of admission for 28 days, or until the date of death, whichever came first. Further exploratory analyses included 14-day outcomes and limiting to second wave data only, acknowledging that these medicines only became available after the first wave.

A propensity score (PS) [18] was created for the treatment comparisons using an inverse probability of treatment weighted model (supplementary 1 in S1 File). This helps account for confounding by indication, a bias introduced when treatment selection in observational studies is influenced by the characteristics of patients. The PS model included the following baseline covariates: age, sex, ethnicity, comorbidity (obesity, diabetes and hypertension), week of admission and admission physiological observations, CRP, neutrophils and chest x-ray abnormality.

The proportional hazards assumption was assessed using Nelson-Aalen plots, plotting Schoenfeld residuals, and testing for time-varying effect of period and treatment in respective analyses (supplementary 2 in S1 File).

The proportion of missing data was small in our variables of interest. The most incomplete data was for Body Mass Index (BMI) (16.8%). Missing data were addressed using multiple imputation with chained equations to generate 20 imputed datasets (supplementary 3 in S1 File). The imputation model involved all variables included in the analyses and was stratified by period to account for potential differences in the association between waves. Results in the unimputed and imputed models were compared. All analyses were undertaken using Stata 16 (StataCorp, USA).

## Results

### COVID admissions

In total 3,949 patients were admitted with COVID-19 during the study period, of which 3,195 (80.9%) were discharged from hospital and 733 (18.6%) have died. Twenty-one patients were still in hospital at the censor date.

**Fig 1:**
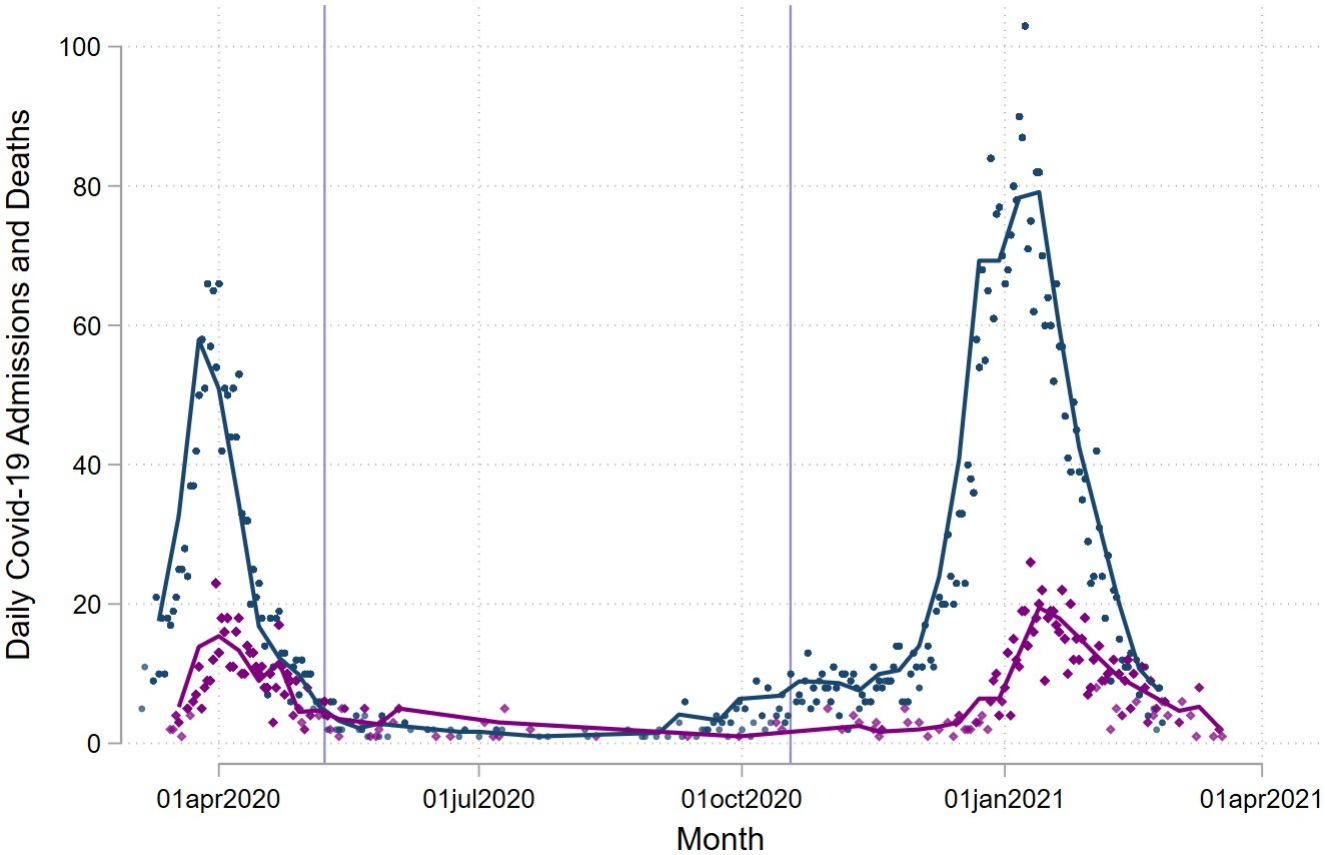
Daily Covid-19 admissions and deaths. Number of daily COVID-19 admissions in blue and 28-day COVID-19 mortality purple by day of admission. Lilac lines represent the end of wave 1 and the beginning of wave 2.

Fig 1 graphically represents the number of daily admissions with two peaks and a trough during the summer months. According to our methods wave 1 was from the 5th March 2020 to the 7th May 2020 and wave 2 from the 18th October 2020 to the 26^th^ February 2021.

### Patient characteristics

More patients were admitted during wave 2. There were striking differences in patient demographics and clinical data at presentation between wave 1 and wave 2 (Table 1). Compared to wave 1, patients admitted during wave 2 were younger (mean age 64 versus 68), less likely to be of Black, Asian or Mixed Ethnicity (BAME) (26.8% versus 34.2%) and less likely to have diabetes (24.3% versus 42.6%) or hypertension (37.0% versus 68.8%). Patients presenting in wave 2 had lower admission neutrophil counts and CRP, with a lower proportion presenting with abnormal chest-x ray findings. Of those with radiographic findings, the x-ray severity score was greater in wave 2 compared to wave 1 (median score 3 [IQR 2–5] versus 4 [IQR 0–6]). There was no difference in the proportion of patients admitted to ICU during both waves. Less than 1 percent (n = 21) of patients admitted during wave 2 remained in hospital at the time of analysis; whilst there are no patients remaining in hospital that were admitted during wave 1.

**Table 1:** Demographics and clinical presentation, by Wave 1 and Wave 2 and period in between two waves.

	Wave 1	Period between Waves	Wave 2	p-value*
Number of patients	N = 1,215	N = 120	N = 2,614	
Age at admission: mean (SD)	68 (17)	66 (20)	64 (18)	<0.001
Sex, Male, n (%)	723 (59.5%)	55 (45.8%)	1,430 (54.7%)	0.005
BAME ethnicity, n (%)	416 (34.2%)	42 (35.0%)	700 (26.8%)	<0.001
Diabetes, n (%)	518 (42.6%)	45 (37.5%)	636 (24.3%)	<0.001
Hypertension, n (%)	836 (68.8%)	76 (63.3%)	967 (37.0%)	<0.001
Obese BMI >/ = 30, n (%)	285 (27.8%)	36 (32.4%)	781 (35.5%)	<0.001
Admission neutrophils, 10^9/L, median (IQR)	6 (4–8)	5 (3–8)	5 (4–7)	<0.001
Admission CRP, mg/L, median (IQR)	86 (41–155)	56 (27–129)	74 (33–132)	<0.001
Physiological observation score, NEWS2**, range 0–20	3 (2–5)	2 (0–4)	3 (1–5)	0.42
Abnormal chest X-ray, n (%)	1,061 (87.8%)	74 (61.7%)	1,492 (69.6%)	<0.001
Chest X-ray score***, median (IQR)	3 (2–5)	5 (3–6)	4 (0–6)	<0.001
Prescription remdesivir, n (%)	40 (3.3%)	19 (15.8%)	837 (32.0%)	<0.001
Prescription dexamethasone, n (%)	18 (1.5%)	30 (25.0%)	1,869 (71.5%)	<0.001
Time to 1st PCR+, days, median, IQR	1 (0–1)	1 (0–1)	0 (0–0)	<0.001
Admitted to ICU, n (%)	194 (16.0%)	18 (15.0%)	429 (16.4%)	0.88
Outcome, n (%): Still admitted	0 (0.0%)	0 (0.0%)	21 (0.8%)	<0.001
Died	332 (27.3%)	18 (15.0%)	383 (14.7%)	
Discharged	883 (72.7%)	102 (85.0%)	2,210 (84.5%)	

* Comparison only between Wave 1 and Wave 2.

** National Early Warning Score (NEWS) 2 is based on a simple aggregate scoring system of 6 physiological parameters: respiration rate, oxygen saturation, systolic blood pressure, pulse rate, level of consciousness or new confusion, temperature. A score is allocated to each parameter as they are measured, with the magnitude of the score reflecting how extremely the parameter varies from the norm. The score is then aggregated and uplifted by 2 points for people requiring supplemental oxygen to maintain their recommended oxygen saturation.

*** Chest radiographs were assessed using an adapted radiographic assessment of lung oedema (RALE) score for COVID-19, as introduced by Wong et al. The severity score attributes a number between 0–4 to each lung depending on extent of consolidation or ground glass opacities (0 = no involvement, 1 = <25%, 2 = 25–49%, 3 = 50–75%, 4 = >75% involvement). Values for each lung were summed to produce a final score ranging from 0–8. Correlation between lungs was high (r = 0.65; κ = 0.44). The first 200 radiographs were assessed by two independent clinicians. Inter-rater concordance demonstrated high agreement (90.5%). Single reading was undertaken for remaining radiographs.

Prescription of remdesivir and dexamethasone was higher during wave 2. Over the entire period patients prescribed either remdesivir (n = 896, 22.7%) or dexamethasone (n = 1917, 48.5%) were more likely to be young, obese, have less comorbidity burden, and present with worse admission physiological observation scores, CRP levels and abnormal chest x-ray findings (Table 2).

**Table 2:** Patient demographics and clinical data at presentation, by remdesivir or by dexamethasone.

	No remdesivir	Remdesivir	p-value	No dexamethasone	Dexamethasone	p-value
Number of patients	N = 2,032	N = 1,917		N = 3,053	N = 896	
Age at admission: mean (SD)	67 (19)	64 (16)	<0.001	67 (18)	61 (15)	<0.001
Sex, Male, n (%)	1,128 (55.5%)	1,080 (56.3%)	0.60	1,692 (55.4%)	516 (57.6%)	0.25
BAME ethnicity, n (%)	633 (31.2%)	525 (27.4%)	0.009	909 (29.8%)	249 (27.8%)	0.25
Diabetes, n (%)	691 (34.0%)	508 (26.5%)	<0.001	961 (31.5%)	238 (26.6%)	0.005
Hypertension, n (%)	1,142 (56.2%)	737 (38.4%)	<0.001	1,540 (50.4%)	339 (37.8%)	<0.001
Obese BMI >/ = 30, n (%)	451 (25.9%)	651 (40.7%)	<0.001	747 (29.0%)	339 (37.8%)	<0.001
Admission neutrophils, 10^9/L, median (IQR)	5 (4–8)	5 (4–7)	0.34	5 (4–8)	5 (4–7)	0.99
Admission CRP, mg/L, median (IQR)	63 (23–126)	90 (51–146)	<0.001	71 (30–133)	94 (56–149)	<0.001
Physiological observation score, NEWS2*, range 0–20	2 (1–4)	4 (2–5)	<0.001	3 (1–4)	4 (3–6)	<0.001
Abnormal chest X-ray, n (%)	1,342 (72.6%)	1,285 (79.1%)	<0.001	1,980 (73.4%)	647 (83.6%)	<0.001
Chest X-ray score***, median (IQR)	3 (2)	4 (3)		3 (3)	5 (3)	<0.001
Prescription of dexamethasone, n (%)	57 (2.8%)	839 (43.8%)	<0.001	-	-	-
Prescription of remdesivir, n (%)	-	-	-	1,078 (35.3%)	839 (93.6%)	<0.001
Time to 1st PCR+, days, median, IQR	0 (0–1)	0 (0–0)	<0.001	0 (0–1)	0 (0–0)	<0.001
Admitted to ICU, n (%)	223 (11.0%)	418 (21.8%)	<0.001	404 (13.2%)	237 (26.5%)	<0.001
Outcome, n (%): Still admitted	3 (0.1%)	18 (0.9%)	<0.001	14 (0.5%)	7 (0.8%)	0.004
Died	401 (19.7%)	332 (17.3%)		599 (19.6%)	134 (15.0%)	
Discharged	1,628 (80.1%)	1,567 (81.7%)		2,440 (79.9%)	755 (84.3%)	

* National Early Warning Score (NEWS) 2 is based on a simple aggregate scoring system of 6 physiological parameters: respiration rate, oxygen saturation, systolic blood pressure, pulse rate, level of consciousness or new confusion, temperature. A score is allocated to each parameter as they are measured, with the magnitude of the score reflecting how extremely the parameter varies from the norm. The score is then aggregated and uplifted by 2 points for people requiring supplemental oxygen to maintain their recommended oxygen saturation.

** Chest radiographs were assessed using an adapted radiographic assessment of lung oedema (RALE) score for COVID-19, as introduced by Wong et al. The severity score attributes a number between 0–4 to each lung depending on extent of consolidation or ground glass opacities (0 = no involvement, 1 = <25%, 2 = 25–49%, 3 = 50–75%, 4 = >75% involvement). Values for each lung were summed to produce a final score ranging from 0–8. Correlation between lungs was high (r = 0.65; κ = 0.44). The first 200 radiographs were assessed by two independent clinicians. Inter-rater concordance demonstrated high agreement (90.5%). Single reading was undertaken for remaining radiographs.

### COVID-19 mortality at 28-days

The 28-day mortality rate was highest during wave 1: 26.1% compared to 13.1% in wave 2 (Table 3). The median time to death was 7 days (IQR 3–12). The competing risk model demonstrated a statistically significant reduction in risk of death in wave 2 compared to the wave 1; [unadjusted SHR 0.47 (95% CI 0.40 to 0.54) p<0.001]. This effect remained in multivariable imputed analysis accounting for variation in age, gender, comorbidity, and COVID-19 severity at presentation [adj SHR 0.49 (95% CI 0.37 to 0.65) p<0.001] (Table 3).

**Table 3:** Cumulative incidence and subhazards of death between Wave 1 and Wave 2 and mortality rate and Cox model hazard of death with remdesivir and dexamethasone.

	Wave 1	Wave 2
Entire period		
Number of patients	1,215	2614
Number of deaths	317	351
28-day mortality rate (%, 95% CI)	26.1 (23.6–28.5)	13.1 (11.8–14.4)
Competing risk model		
Unadjusted SHR	Ref	0.47 (0.40, 0.54) †
Fully adjusted (imputed) SHR	Ref	0.49 (0.37, 0.65) †
	No Remdesivir	Remdesivir
Entire period		
Number of patients	3053	896
Number of deaths	571	114
28-day mortality rate (%)	18.7 (17.2–20.3)	12.7 (10.5–15.3)
Cox model unadjusted HR	Ref	0.64 (0.53, 0.79) †
Cox model propensity imputed HR*	Ref	0.84 (0.65, 1.08)
Limited to wave 2		
Number of patients	1777	837
Number of deaths	244	107
28-day mortality rate (%)	13.7 (12.1–15.6)	12.4 (10.5–15.4)
Cox model unadjusted HR	Ref	0.91 (0.73, 1.14)
Cox model propensity imputed HR*	Ref	1.23 (0.94, 1.62)
	No Dexamethasone	Dexamethasone
Entire period		
Number of patients	2032	1917
Number of deaths	384	301
28-day mortality rate (%)	18.9 (17.1–20.1)	15.7 (14.0–17.6)
Cox model unadjusted HR	Ref	0.80 (0.69, 0.93) ^#^
Cox model propensity imputed HR*	Ref	0.97 (0.70, 1.35)
Limited to wave 2		
Number of patients	745	1869
Number of deaths	60	291
28-day mortality rate (%)	8.1 (6.1–10.4)	15.6 (13.8–17.5)
Cox model unadjusted HR	Ref	1.99 (1.50, 2.62) ^†^
Cox model propensity imputed HR*	Ref	1.05 (0.69, 1.59)

P values ^†^ = <0.001.

* Adjusted age, sex, ethnicity, hypertension, diabetes obesity and baseline physiological observation score, CRP, neutrophil, chest x-ray abnormality, remdesivir and dexamethasone. P values † = <0.001

* Adjusted age, sex, ethnicity, hypertension, diabetes obesity and baseline physiological observation score, CRP, Neutrophil, chest x-ray abnormality, remdesivir and dexamethasone.

**Fig 2:**
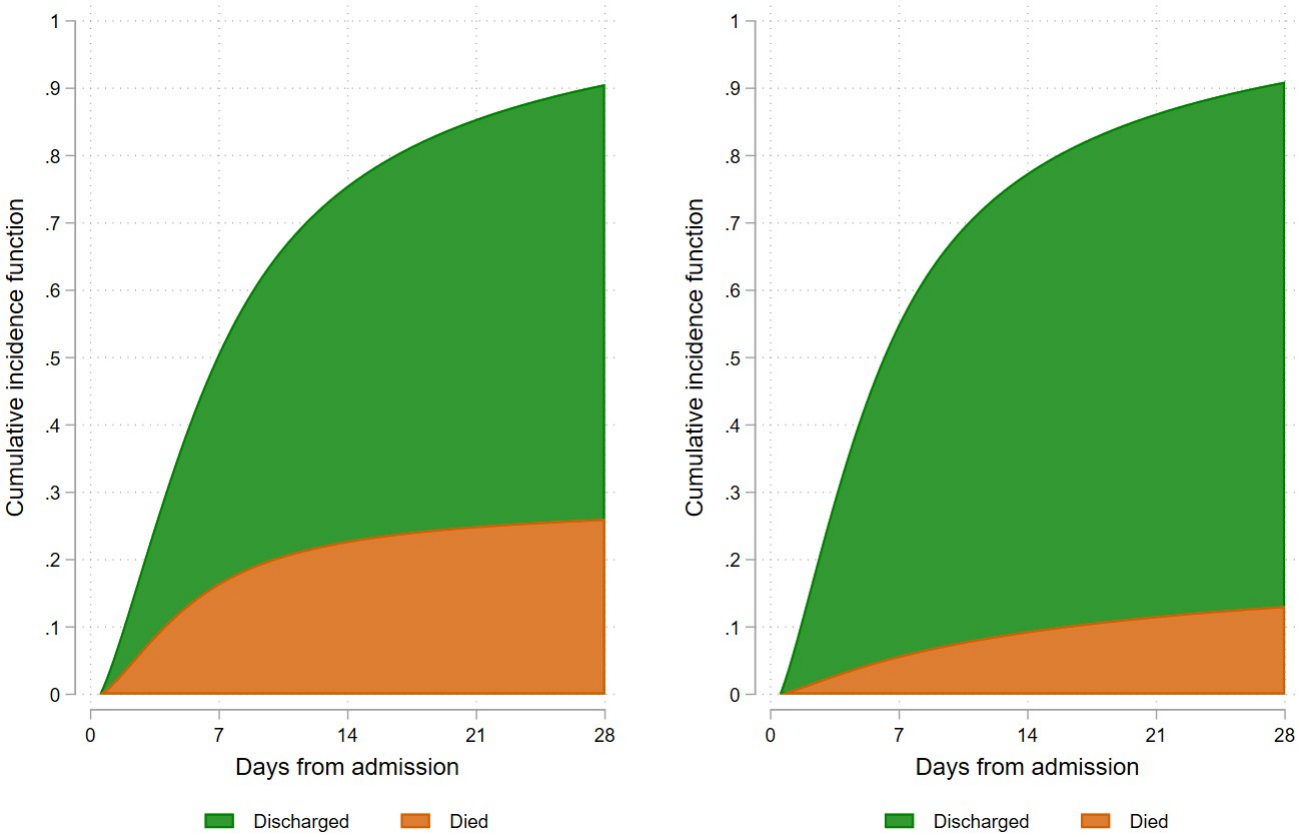
Cumulative incidence of COVID-19 death.

When proportionality assumptions from the competing-risks survival model were reviewed, it was clear the risk of death during the period of admission changed over time (supplementary 2 in S1 File). The majority of the increased mortality risk was in the first 7 to 14 days. In the wave 1, the cumulative incidence of death was 16.5% by day 7, 22.8% by day 14, 25.0% by day 21 and 26.1% by day 28. These compared to 5.7%, 9.4%, 11.6% and 13.1% in wave 2 respectively. Results are displayed graphically in Fig 2.

### Impact of remdesivir and dexamethasone

The 28-day mortality rate was lower in patients prescribed remdesivir compared to those not prescribed remdesivir (12.7% versus 18.7%), and in patients prescribed dexamethasone compared to those not prescribed dexamethasone (15.7% versus 18.9%) (Table 3). The use of remdesivir associated with a statistically significant lower mortality [unadjusted HR 0.64 (0.53, 0.79), p<0.001], although the difference was no longer significant in the propensity adjusted model [imputed propensity HR (adjHR) 0.84 (95% CI 0.65 to 1.08), p = 0.17]. For dexamethasone the magnitude of benefit was numerically smaller than for remdesivir, and again, statistically significant only in the unadjusted model; [HR 0.80 (95% CI 0.69 to 0.93), p = 0.003 and adjHR 0.97 (95% CI 0.70 to 1.35), p = 0.87].

The model diagnostics demonstrated that Cox proportionality assumptions were violated in both the remdesivir and dexamethasone models. Sensitivity analyses examining time ‘at risk’ from the date of admission until day 14 days, during which the Cox model assumptions were not violated, demonstrated that the adjusted HR remained significant (in favour of a benefit) for remdesivir [adjHR 0.58 (95% CI 0.42 to 0.79), p = 0.001] but not dexamethasone [adjHR 0.90 (95% CI 0.62 to 1.32), p = 0.60] (supplementary 4 in S1 File).

Evaluation of the propensity models identified significant imbalance between cohorts, both for remdesivir and dexamethasone (supplementary 1 in S1 File). Remdesivir and dexamethasone were not prescribed in the first wave. We therefore undertook analyses limited to wave 2 only.

### Impact of remdesivir and dexamethasone in wave 2

Limiting our analyses to Wave 2 demonstrates a lower 28-day mortality rate in patients prescribed remdesivir compared to those not prescribed remdesivir (12.4% versus 13.7%), and a higher 28-day mortality rate in patients prescribed dexamethasone compared to those not prescribed dexamethasone (15.6% versus 8.1%) (Table 3). The propensity model diagnostics are better, with reasonable balance [supplement 1 in S1 File]. In Wave 2, remdesivir did not reach statistical significance in the unadjusted analysis [HR 0.91 (95% CI 0.73 to 1.14) p = 0.42] or in the imputed propensity model [HR 1.23 (95% CI 0.94 to 1.62) p = 0.13]. Dexamethasone associated with an increase in mortality risk in the unadjusted analysis [HR 1.99 (95% CI 1.50 to 2.62), p<0.001], but this finding did not persist in imputed propensity modelling [HR 1.05 (95% CI 0.69 to 1.59), p = 0.83] (Table 3).

## Discussion

Our analysis has identified striking reductions in mortality among patients admitted to hospital with virologically confirmed Covid-19 between March 2020 and January 2021. We observed significant differences between the patients admitted across the two waves. The risk of dying in a patient admitted with COVID-19 was more than 50% lower in the second wave compared to the first. We have been unable to explain the improvement in survival by changing population or disease severity, as even after adjusting for demographics, comorbidity, and measures of physiological disturbance the differences remained. In our analyses, neither dexamethasone nor remdesivir appeared to be key explanatory variables either. Potential explanations include changes in the viral pathogenicity, changes in the phenotype of the patients exposed, or changes in the care pathway that we have not captured.

### Could COVID-19 virulence explain the reduction in mortality?

In November 2020 a new mutation (VOC 202012/01) appeared in the South East of England. The hospitals in this study are situated in the south east of England and it is likely that the majority of patients studied in wave 2 had the new strain [19]. A community based analysis from Public Health England suggested that the new strain was more transmissible but was not associated with increased mortality. The UK Scientific Advisory Group for Emergencies (SAGE) minutes hint that evidence may be emerging for a worse outcome for the new variant [20]. We do not have data on the SARS-Cov-2 subtypes in our cohort but our data show that early hospital outcomes for Covid-19 were better when the VOC 202012/01 variant was prevalent in the local community.

### Could changing patient populations explain the reduction in mortality?

There were differences between patients admitted in wave 1 and 2. Patients in wave 2 were younger, less likely to be from an ethnic minority or have a history of diabetes or hypertension. BAME groups were heavily over-represented in the first wave [21]. Although there is a clear excess of disease amongst the BAME community there were no differences in survival once in hospital [3]. Why ethnic minorities formed a smaller proportion of admissions during the second wave is unclear; possibilities include changes in behaviour or that patients exposed in wave 1 are now immune [22, 23]. Inflammatory markers were lower in wave 2 but there was no difference in physiological observation scores, which predict risk of early deterioration or death [24]. The lower mortality risk in wave 2 persists after adjusting for age, ethnicity, comorbidity and admission disease severity. Although we may not have evaluated all possible risk factors for death with the potential for unmeasured confounding, our results suggest that differences in the medical management of COVID-19 between the two waves may have resulted in improved survival. National UK audit data has demonstrated much smaller improvement in COVID-19 outcomes for patients admitted to critical care [25].

### Could changes in care pathways have explained the reduction in mortality?

Better admission and discharge decisions: Analysis of patients admitted in the first wave led to the development of risk stratification scores, assisting with admission and discharge decisions with early identification of patients at high risk of clinical deterioration [3, 4]. Improved supportive care: An appreciation that patients with severe COVID-19 are hypercoagulable led to more aggressive anticoagulation strategies, as well as a lower threshold for CT pulmonary angiography [26]. Strengthened NHS infrastructure: Use of non-invasive ventilation (NIV) and high flow oxygen devices were initially limited due to equipment and skill shortages, limitations on space to undertake aerosol generating procedures, as well as shortage of oxygen supply. In the 2^nd^ wave, hospitals were more prepared. These changes were captured in robust national guidelines, that were published in a staggered fashion during the first wave, enabling a more evidence-based approach for the second wave [27].

### How much impact did remdesivir and dexamethasone have?

Another key difference in clinical care in the first and second wave has been the use of dexamethasone and remdesivir following positive results from RCTs [5, 6]. Our analyses examined whether any differences in survival were attributable to either drug. It is crucial to interpret our findings with an awareness that they are from real world data, and there is likely to be substantial unmeasured confounding attributable to the selection of patients for treatment. We separately examined the whole period and also only the period after both drugs were introduced into routine care to reduce confounding from other management strategies that have changed over time.

The analysis using data from across the entire period showed a statistically significant association between remdesivir and lower mortality. Important characteristics differed between people receiving remdesivir and those not. For example, people receiving remdesivir had a baseline physiological observation score double that of patients not prescribed remdesivir, suggesting channelling of use towards more severe disease. We used a propensity model to understand effects of the drug after accounting for disease severity. After adjustment, the association was only significant for the day 14 analysis, which is similar to the findings from the ACTT-1 trial [6]. When limited to Wave 2 only, where the propensity model diagnostics were superior, remdesivir did not associate with a statistically significant reduction in 14 or 28-day mortality. Based on the results, we would conclude that remdesivir is not the main explanatory factor for the reduction in mortality between wave 1 and wave 2, although we cannot exclude that it has some meaningful effect.

For dexamethasone, analysis of the entire cohort demonstrated a survival advantage, which lost significance after adjustment. Limited to Wave 2 only, there was a paradoxical appearance of harm with dexamethasone, which disappeared when accounting for patient factors and disease severity. These findings are not incompatible with Recovery trial, with confidence intervals from our adjusted model overlapping with the effect observed in the trial (HR 0.83, 95% CI 0.75, 0.93) [5]. It is difficult to draw robust conclusions from the dexamethasone analysis as unmeasured confounding is likely to be a major factor. Perhaps the most important finding is to highlight the challenges of interrogating real-world cohorts to evaluate drug effectiveness.

There are important limitations to consider. It is possible that SARS CoV-2 testing was not applied equally in wave 1 and wave 2, particularly in less severely ill patients, accounting for an underreporting of milder COVID-19 diagnoses. By wave 2 a greater number of SARS CoV-2 tests were performed nationally, and case capture (especially for milder cases) may have been superior. There are possible methodological flaws in the extraction of comorbidity data, however this was applied in the same way across both waves and should not influence our results. The reporting of diabetes and hypertension on admission may have increased due to the recognised association with COVID-19 outcomes. This may have been greater in wave 2. We have not controlled for concomitant medication, or patient participation in RCTs. During the time window, there was little use of tocilizumab through routine care, but again these data were not available for analysis. For both remdesivir and dexamethasone analyses we did not have data on oxygen saturation on air on admission, and so it as not possible to determine eligibility for treatment (in the UK both drugs are only recommended for patients requiring supplemental oxygen to maintain saturations >94%).

This study benefits from a large prospective cohort with relatively complete data. The sampled population from both waves had a high disease burden, and the findings represent outcomes from a real-world setting. We have been able to include detailed confounders in our modelling, allowing robust adjustment for disease severity as well as patient demographics. Our sub-analysis of remdesivir and dexamethasone should not be considered as true efficacy data but should stimulate further observational research using large regional or national cohorts. What is clear from our analyses is that the substantial improvement in mortality is not due to dexamethasone and remdesivir alone.

## Conclusion

We have demonstrated a substantial improvement in early mortality for patients hospitalised with virologically confirmed Covid-19, and a striking change in the characteristics of patients admitted. We could not confirm the benefits of remdesivir or dexamethasone. The improvements in survival appear unlikely to be attributable to the introduction of these drugs alone. Potential explanations for the improved outcomes may lie in factors we did not measure, such as better admission and discharge decisions, improved supportive care and strengthened healthcare infrastructure. Future research in this field should investigate these, along with other causes for improvement in survival. These may include analyses of COVID-19 variants or vaccination status and immune response in hospitalised patients.

## Supporting information

S1 File (DOCX)
