# Supplementary material for: Inpatient COVID-19 mortality has reduced over time: Results from an observational cohort
Source: PLoS One. 2022 Jan 13;17(1):e0261142. doi: 10.1371/journal.pone.0261142 (PMC8757902; doi:10.1371/journal.pone.0261142)
Supplement: S1 File — (DOCX) [file pone.0261142.s001.docx]

**Table of Contents for Supplementary Figures and Tables**

[Supplementary 1. Propensity model methodology 2](#_Toc71809722)

[Figure 1a. histogram of propensity score for remdesivir i) entire period complete case & ii) entire period imputed iii) wave 2 complete case & ii) wave 2 imputed 2](#_Toc71809723)

[Table 1b. Comparison of baseline covariates in weighted cohorts with remdesivir for entire period. 3](#_Toc71809724)

[Table 1c. Comparison of baseline covariates in weighted cohorts with remdesivir for wave 2 4](#_Toc71809725)

[Figure 1d. histogram of propensity score for dexamethasone i) entire period complete case & ii) entire period imputed iii) wave 2 complete case & ii) wave 2 imputed 5](#_Toc71809726)

[Table 1e. Comparison of baseline covariates in weighted cohorts with dexamethasone for entire period. 6](#_Toc71809727)

[Table 1f. Comparison of baseline covariates in weighted cohorts with dexamethasone for wave 2 7](#_Toc71809728)

[Supplementary 2: Proportionality assumptions 8](#_Toc71809729)

[Supplementary 2a. Plotted hazard estimates demonstrate non-proportionality in Wave 1 versus Wave 2 8](#_Toc71809730)

[analysis as indicated by non-parallel smoothed hazard function. 8](#_Toc71809731)

[Supplementary 2b: Cumulative incidence and mortality rate at 7, 14, 21 and 28 days 9](#_Toc71809732)

[Supplementary 2c: Plotted hazard estimates demonstrate non-proportionality in remdesivir analysis as indicated by non-parallel smoothed hazard function. 9](#_Toc71809733)

[Supplementary 2d: Plotted hazard estimates demonstrate non-proportionality in dexamethasone analysis as indicated by non-parallel smoothed hazard function. 10](#_Toc71809734)

[Supplementary 3. Imputation methodology 11](#_Toc71809735)

[Table 3a. Missing data 11](#_Toc71809736)

[Figure 3b. Imputation diagnostics 12](#_Toc71809737)

[Supplementary 4. Full results for Wave and drug analyses 13](#_Toc71809738)

[Table 4a. Cumulative incidence and competing risk hazard of death between the three period 13](#_Toc71809739)

[Table 4b. Mortality rate and Cox proportional model hazard of death with Remdesivir 14](#_Toc71809740)

[Table 4c. Mortality rate and Cox proportional model hazard of death with Dexamethasone 15](#_Toc71809741)

[Figure 4d. Kaplan Meier survival estimate, i) Remdesivir and ii) Dexamethasone 16](#_Toc71809742)

# **Supplementary 1. Propensity model methodology**

A multivariable logistic regression model using identify baseline covariates that predicted treatment choice (model 1: remdesivir versus no remdesivir and model 2: dexamethasone versus no dexamethasone) was used to create a single propensity score for each individual.

The best model was chosen using the Hosmer-Lemeshow goodness of fit test. A propensity score model was created including the following covariates: age, gender, week of admission, diabetes hypertension, obesity, BAME, CRP, neutrophil, first physiological observation score and chest x-ray abnormality. K density plots and histograms were used to graphically demonstrate differences in propensity scores between the treatment groups

The inverse of the probability (or the inverse of 1 minus the probability in the no treatment group) was then used as the treatment weight in the analysis. Truncation of weights was used to prevent a small number of larger weights de-stabilising the model. The balancing of the cohorts using the weighted model was tested by comparing standardised differences between cohorts. The weighted means and standard differences are shown below.

## **Figure 1a. histogram of propensity score for remdesivir i) entire period complete case & ii) entire period imputed iii) wave 2 complete case & ii) wave 2 imputed**

****i) ii)

iii) iv)

## **Table 1b. Comparison of baseline covariates in weighted cohorts with remdesivir for entire period.**

|  | Mean in treated | Mean in untreated | Standardised difference |
| --- | --- | --- | --- |
| **Unbalanced** | | | |
| Week | 3169.04 | 3151.7 | 1.12 |
| Age by decade | 6.18 | 6.86 | -0.419 |
| Sex | 1.57 | 1.55 | 0.04 |
| Diabetes | 0.32 | 0.34 | -0.037 |
| Hypertension | 0.44 | 0.53 | -0.174 |
| Obesity | 0.45 | 0.28 | 0.36 |
| Ethnicity: Asian | 0.05 | 0.04 | 0.066 |
| Black | 0.2 | 0.24 | -0.089 |
| Mixed | 0.02 | 0.01 | 0.084 |
| Missing | 0.17 | 0.12 | 0.143 |
| Other | 0.09 | 0.05 | 0.135 |
| White | 0.46 | 0.54 | -0.146 |
| CRP | 109.36 | 90.47 | 0.238 |
| Neutrophils | 5.90 | 6.19 | -0.074 |
| NEWs score | 4.09 | 2.91 | 0.506 |
| CXR total | 4.63 | 2.97 | 0.602 |
| **Balanced complete case** | | | |
| Week | 3162.16 | 3155.54 | 0.428 |
| Age by decade | 6.45 | 6.72 | -0.164 |
| Sex | 1.58 | 1.55 | 0.063 |
| Diabetes | 0.33 | 0.33 | 0 |
| Hypertension | 0.51 | 0.51 | -0.006 |
| Obesity | 0.35 | 0.32 | 0.072 |
| Ethnicity: Asian | 0.05 | 0.04 | 0.038 |
| Black | 0.25 | 0.23 | 0.051 |
| Mixed | 0.01 | 0.01 | -0.01 |
| Missing | 0.14 | 0.13 | 0.022 |
| Other | 0.08 | 0.07 | 0.071 |
| White | 0.46 | 0.52 | -0.108 |
| CRP | 98.64 | 94.66 | 0.05 |
| Neutrophils | 6.09 | 6.12 | -0.008 |
| NEWs score | 3.50 | 3.18 | 0.136 |
| CXR total | 3.71 | 3.35 | 0.130 |
| **Balanced imputed** | | | |
| Week | 3164.98 | 3157.28 | 0.522 |
| Age by decade | 6.55 | 6.76 | -0.131 |
| Sex | 1.57 | 1.55 | 0.044 |
| Diabetes | 0.36 | 0.39 | -0.068 |
| Hypertension | 0.55 | 0.6 | -0.106 |
| Obesity | 0.36 | 0.33 | 0.071 |
| Ethnicity: Asian | 0.05 | 0.05 | 0.001 |
| Black | 0.29 | 0.27 | 0.045 |
| Mixed | 0.02 | 0.02 | 0.003 |
| Other | 0.1 | 0.08 | 0.06 |
| White | 0.55 | 0.58 | -0.076 |
| CRP | 95.55 | 96.09 | -0.007 |
| Neutrophils | 5.86 | 5.93 | -0.016 |
| NEWs score | 3.33 | 3.34 | -0.004 |
| CXR total | 3.75 | 3.43 | 0.116 |
| Week | 3164.98 | 3157.28 | 0.522 |

## **Table 1c. Comparison of baseline covariates in weighted cohorts with remdesivir for wave 2**

|  | Mean in treated | Mean in untreated | Standardised difference |
| --- | --- | --- | --- |
| **Unbalanced** | | | |
| Week | 3171.51 | 3171.2 | 0.109 |
| Age by decade | 6.2 | 6.71 | -0.308 |
| Sex | 1.56 | 1.53 | 0.063 |
| Diabetes | 0.32 | 0.32 | -0.001 |
| Hypertension | 0.43 | 0.5 | -0.122 |
| Obesity | 0.46 | 0.29 | 0.356 |
| Ethnicity: Asian | 0.05 | 0.04 | 0.038 |
| Black | 0.19 | 0.21 | -0.035 |
| Mixed | 0.02 | 0.01 | 0.076 |
| Missing | 0.17 | 0.14 | 0.082 |
| Other | 0.48 | 0.54 | -0.121 |
| White | 0.09 | 0.06 | 0.103 |
| CRP | 110.12 | 82.18 | 0.371 |
| Neutrophils | 5.84 | 5.85 | -0.005 |
| NEWs score | 4.11 | 2.80 | 0.577 |
| CXR total | 4.74 | 3.05 | 0.576 |
| **Balanced complete case** | | | |
| Week | 3171.39 | 3171.32 | 0.025 |
| Age by decade | 6.58 | 6.55 | 0.02 |
| Sex | 1.56 | 1.54 | 0.043 |
| Diabetes | 0.33 | 0.32 | 0.021 |
| Hypertension | 0.49 | 0.48 | 0.023 |
| Obesity | 0.37 | 0.35 | 0.036 |
| Ethnicity: Asian | 0.05 | 0.05 | -0.002 |
| Black | 0.21 | 0.2 | 0.027 |
| Mixed | 0.01 | 0.01 | -0.008 |
| Missing | 0.13 | 0.14 | -0.034 |
| Other | 0.52 | 0.52 | -0.003 |
| White | 0.08 | 0.07 | 0.015 |
| CRP | 94.8 | 91.74 | 0.041 |
| Neutrophils | 5.76 | 5.82 | -0.016 |
| NEWs score | 3.36 | 3.27 | 0.04 |
| CXR total | 3.90 | 3.66 | 0.082 |
| **Balanced imputed** | | | |
| Week | 3171.46 | 3171.24 | 0.081 |
| Age by decade | 6.65 | 6.67 | -0.01 |
| Sex | 1.56 | 1.53 | 0.048 |
| Diabetes | 0.34 | 0.34 | 0 |
| Hypertension | 0.51 | 0.52 | -0.009 |
| Obesity | 0.36 | 0.35 | 0.003 |
| Ethnicity: Asian | 0.05 | 0.06 | -0.017 |
| Black | 0.24 | 0.23 | 0.031 |
| Mixed | 0.02 | 0.02 | -0.001 |
| Other | 0.09 | 0.09 | 0.007 |
| White | 0.6 | 0.61 | -0.023 |
| CRP | 91.94 | 90.46 | 0.02 |
| Neutrophils | 5.71 | 5.77 | -0.016 |
| NEWs score | 3.13 | 3.32 | -0.083 |
| CXR total | 3.85 | 3.62 | 0.077 |

## **Figure 1d. histogram of propensity score for dexamethasone i) entire period complete case & ii) entire period imputed iii) wave 2 complete case & ii) wave 2 imputed**

****i) ii)

iii) iv)

## **Table 1e. Comparison of baseline covariates in weighted cohorts with dexamethasone for entire period.**

|  | Mean in treated | Mean in untreated | Standardised difference |
| --- | --- | --- | --- |
| **Unbalanced** | | | |
| Week | 3170.67 | 3143.2 | 2.085 |
| Age by decade | 6.5 | 6.89 | -0.233 |
| Sex | 1.56 | 1.54 | 0.026 |
| Diabetes | 0.33 | 0.33 | -0.01 |
| Hypertension | 0.47 | 0.54 | -0.156 |
| Obesity | 0.4 | 0.25 | 0.317 |
| Ethnicity: Asian | 0.05 | 0.04 | 0.029 |
| Black | 0.21 | 0.25 | -0.101 |
| Mixed | 0.02 | 0.01 | 0.084 |
| Missing | 0.16 | 0.11 | 0.133 |
| Other | 0.07 | 0.05 | 0.07 |
| White | 0.5 | 0.54 | -0.071 |
| CRP | 105.66 | 85.63 | 0.248 |
| Neutrophils | 5.99 | 6.24 | -0.065 |
| NEWs score | 3.83 | 2.64 | 0.5 |
| CXR total | 4.3 | 2.56 | 0.652 |
| **Balanced complete case** | | | |
| Week | 3159.42 | 3155.14 | 0.325 |
| Age by decade | 6.66 | 6.84 | -0.105 |
| Sex | 1.53 | 1.52 | 0.021 |
| Diabetes | 0.33 | 0.34 | -0.03 |
| Hypertension | 0.47 | 0.52 | -0.097 |
| Obesity | 0.37 | 0.3 | 0.146 |
| Ethnicity: Asian | 0.03 | 0.06 | -0.152 |
| Black | 0.23 | 0.24 | -0.017 |
| Mixed | 0.01 | 0.01 | 0.027 |
| Missing | 0.12 | 0.11 | 0.009 |
| Other | 0.07 | 0.05 | 0.103 |
| White | 0.54 | 0.53 | 0.014 |
| CRP | 90.26 | 91.74 | -0.018 |
| Neutrophils | 6.02 | 5.96 | 0.015 |
| NEWs score | 3.09 | 3.09 | 0.001 |
| CXR total | 3.35 | 3.27 | 0.032 |
| **Balanced imputed** | | | |
| Week | 3163.18 | 3157.21 | 0.451 |
| Age by decade | 6.68 | 6.88 | -0.121 |
| Sex | 1.54 | 1.52 | 0.058 |
| Diabetes | 0.37 | 0.43 | -0.115 |
| Hypertension | 0.53 | 0.64 | -0.213 |
| Obesity | 0.38 | 0.32 | 0.129 |
| Ethnicity: Asian | 0.04 | 0.08 | -0.183 |
| Black | 0.27 | 0.27 | 0.009 |
| Mixed | 0.02 | 0.01 | 0.058 |
| Other | 0.09 | 0.06 | 0.108 |
| White | 0.58 | 0.58 | -0.001 |
| CRP | 93.85 | 91.45 | 0.030 |
| Neutrophils | 5.94 | 5.62 | 0.086 |
| NEWs score | 3.08 | 3.19 | -0.046 |
| CXR total | 3.57 | 3.6 | -0.012 |

**Table 1f. Comparison of baseline covariates in weighted cohorts with dexamethasone for wave 2**

|  | Mean in treated | Mean in untreated | Standardised difference |
| --- | --- | --- | --- |
| **Unbalanced** | | | |
| Week | 3171.58 | 3170.54 | 0.336 |
| Age by decade | 6.49 | 6.67 | -0.095 |
| Sex | 1.56 | 1.48 | 0.168 |
| Diabetes | 0.33 | 0.3 | 0.065 |
| Hypertension | 0.47 | 0.5 | -0.068 |
| Obesity | 0.4 | 0.22 | 0.381 |
| Ethnicity: Asian | 0.05 | 0.04 | 0.014 |
| Black | 0.2 | 0.2 | 0.018 |
| Mixed | 0.02 | 0.01 | 0.11 |
| Missing | 0.16 | 0.13 | 0.085 |
| Other | 0.07 | 0.07 | 0.028 |
| White | 0.5 | 0.56 | -0.12 |
| CRP | 105.66 | 53.09 | 0.752 |
| Neutrophils | 5.97 | 5.51 | 0.127 |
| NEWs score | 3.85 | 1.6 | 1.077 |
| CXR total | 4.3 | 1.75 | 0.948 |
| **Balanced complete case** | | | |
| Week | 3171.25 | 3171.41 | -0.053 |
| Age by decade | 6.65 | 6.71 | -0.033 |
| Sex | 1.54 | 1.51 | 0.076 |
| Diabetes | 0.31 | 0.34 | -0.059 |
| Hypertension | 0.49 | 0.5 | -0.021 |
| Obesity | 0.35 | 0.32 | 0.052 |
| Ethnicity: Asian | 0.04 | 0.09 | -0.215 |
| Black | 0.2 | 0.21 | -0.04 |
| Mixed | 0.02 | 0.01 | 0.055 |
| Missing | 0.15 | 0.12 | 0.081 |
| Other | 0.08 | 0.05 | 0.125 |
| White | 0.52 | 0.52 | -0.011 |
| CRP | 90.05 | 89.56 | 0.007 |
| Neutrophils | 5.77 | 5.46 | 0.085 |
| NEWs score | 3.2 | 3.21 | -0.006 |
| CXR total | 3.61 | 3.64 | -0.014 |
| **Balanced imputed** | | | |
| Week | 3171.5 | 3170.78 | 0.233 |
| Age by decade | 6.69 | 6.8 | -0.064 |
| Sex | 1.54 | 1.5 | 0.093 |
| Diabetes | 0.32 | 0.39 | -0.154 |
| Hypertension | 0.49 | 0.55 | -0.112 |
| Obesity | 0.35 | 0.34 | 0.006 |
| Ethnicity: Asian | 0.05 | 0.09 | -0.188 |
| Black | 0.24 | 0.24 | 0.002 |
| Mixed | 0.02 | 0.01 | 0.082 |
| Other | 0.08 | 0.08 | 0.021 |
| White | 0.61 | 0.58 | 0.053 |
| CRP | 89.11 | 85.08 | 0.06 |
| Neutrophils | 5.81 | 5.26 | 0.152 |
| NEWs score | 3.15 | 3.16 | -0.006 |
| CXR total | 3.52 | 3.98 | -0.168 |

# **Supplementary 2: Proportionality assumptions**

## **Supplementary 2a. Plotted hazard estimates demonstrate non-proportionality in Wave 1 versus Wave 2** **analysis as indicated by non-parallel smoothed hazard function.**

Probability of death

Test of proportional-hazards assumption indicates violation (Chi 14.4 p value 0.0001). Schoenfeld residual demonstrate non-proportionality with residuals increase over time. Including wave as a time varying covariate confirms increasing hazard over time for wave 2 relative to wave 1; sHR 1.06 (95% CI 1.04-1.09) p = <0.001

## **Supplementary 2b: Cumulative incidence and mortality rate at 7, 14, 21 and 28 days**

As the proportional-hazards assumption wasn’t met, we estimated cumulative incidence to account for this; stratifying analysis by wave and thus allowing for separate baseline hazard function estimates (figure 2).

The cumulative incidence mortality rates (95% CI) at 7, 14, 21 and 28 days are as follows:

| Day | Wave 1 | Wave 2 |
| --- | --- | --- |
| 7 | 16.5% (14.6%- 18.5%) | 5.7% (4.9%-6.6%) |
| 14 | 22.8% (20.5%- 25.1%) | 9.4% (8.3%-10.5%) |
| 21 | 25.0% (22.6%- 27.4%) | 11.6% (10.4%-12.8%) |
| 28 | 26.1% (23.6%- 28.5%) | 13.1% (11.8%-14.4%) |

## **Supplementary 2c: Plotted hazard estimates demonstrate non-proportionality in remdesivir analysis as indicated by non-parallel smoothed hazard function.**

Probability of death

Test of proportional-hazards assumption indicates violation (Chi 23.8, p value <0.001). Schoenfeld residual demonstrating non-proportionality with residuals increase over time. Including remdesivir as a time varying covariate confirms increasing hazard over time for those receiving remdesivir relative to those not receiving remdesivir; HR 1.09 (95% CI 1.06-1.12) p = <0.001

## **Supplementary 2d: Plotted hazard estimates demonstrate non-proportionality in dexamethasone analysis as indicated by non-parallel smoothed hazard function.**

Probability of death

Test of proportional-hazards assumption indicates violation (Chi 28.9, p value <0.001). Schoenfeld residual demonstrating non-proportionality with residuals increase over time. Including dexamethasone as a time varying covariate confirms increasing hazard over time for those receiving dexamethasone relative to those not receiving dexamethasone; HR 1.08 (95% CI 1.05, 1.10) p = <0.001

# **Supplementary 3. Imputation methodology**

Missing data were addressed using multiple imputation. There were missing data for several variables used in the multivariate analysis and in the propensity score. Missing data are presented in table 3a.

These variables with incomplete data were imputed. All missing data was imputed regardless of the reason or reasons it was missing. Separate imputations were performed for Wave 1 and Wave 2 and the period inbetween. The following variables with complete data were utilised for the imputation: age; gender; diabetes; hypertension, number of prescribes drugs, remdesivir, dexamethasone and outcome data with time to outcome. Linear regression, logistic regression and predictive mean matching were performed to impute the normally distributed, dichotomous and non-normally distributed variables respectively.

The data were imputed using multivariate sequential imputation using chained equations. Firstly, all missing values were filled in by simple random sampling with replacement from the observed values. The first variable with missing values, was regressed on all other variables. The imputation was 20 cycles, where at the end of the cycle one imputed dataset was created and the process was repeated to create 20 imputed datasets.

Diagnostic plots were performed and examined for each imputed variable. The 20 datasets were combined using Rubin’s rules therefore, the estimates and standard errors presented here are the combined ones.

## **Table 3a. Missing data**

| Variable | Observations at baseline | Missing Variables |
| --- | --- | --- |
| Ethnicity | 3323 | 626 |
| Obesity | 3337 | 612 |
| Admission neutrophil | 3905 | 44 |
| Admission CRP | 3844 | 105 |
| Admission physiological observation score | 3822 | 127 |
| Chest X-ray score | 3297 | 652 |
| Abnormality on chest X-ray | 3472 | 477 |

## **Figure 3b. Imputation diagnostics**

# **Supplementary 4. Full results for Wave and drug analyses**

## **Table 4a. Cumulative incidence and competing risk hazard of death between the two waves**

|  | Wave 1  (n=1,215) | Wave 2  (n=2614) |
| --- | --- | --- |
| 28 day |  |  |
| Number of deaths | 317 | 351 |
| Unadjusted SHR | Ref | 0.47 (0.40, 0.54) † |
| Age and sex adjusted SHR | Ref | 0.55 (0.48, 0.65) † |
| Fully adjusted SHR | Ref | 0.39 (0.28, 0.55) † |
| Fully adjusted (imputed) SHR | Ref | 0.49 (0.37, 0.65) † |
| 14 day |  |  |
| Number of deaths 14 days | 284 | 265 |
| Unadjusted SHR | Ref | 0.40 (0.34, 0.47) † |
| Fully adjusted SHR | Ref | 0.41 (0.28, 0.61) † |
| Fully adjusted (imputed) SHR | Ref | 0.51 (0.37, 0.69) † |

** Adjusted age, sex, ethnicity, hypertension, diabetes obesity and baseline physiological observation score, CRP, neutrophil, chest x-ray abnormality, remdesivir and dexamethasone.* P values ^†^= <0.001

## **Table 4b. Mortality rate and Cox proportional model hazard of death with Remdesivir**

|  | No Remdesivir | Remdesivir |
| --- | --- | --- |
| Entire period of data collection - 28 day | n=3053 | n=896 |
| Number of deaths 28 days | 571 | 114 |
| Unadjusted HR | Ref | 0.64 (0.53, 0.79) † |
| Fully adjusted HR | Ref | 0.83 (0.63, 1.09) |
| Propensity imputed HR | Ref | 0.84 (0.65, 1.08) |
| Entire period of data collection - 14 day | n=3053 | n=896 |
| Number of deaths 14 days | 493 | 72 |
| Unadjusted HR | Ref | 0.47 (0.37, 0.61) † |
| Fully adjusted HR | Ref | 0.65 (0.47, 0.91) * |
| Propensity imputed HR | Ref | 0.58 (0.42, 0.79) † |
| Limited to Wave 2 - 28 day | n=1777 | n=837 |
| Number of deaths 28 days | 244 | 107 |
| Unadjusted HR | Ref | 0.91 (0.73, 1.14) |
| Fully adjusted HR 28 days | Ref | 0.98 (0.73, 1.31) |
| Propensity imputed HR 28 days | Ref | 1.23 (0.94, 1.62) |
| Limited to Wave 2 - 14 day | n=1777 | n=837 |
| Number of deaths 14 days | 197 | 68 |
| Unadjusted HR | Ref | 0.72 (0.55, 0.95) * |
| Fully adjusted HR | Ref | 0.78 (0.55, 1.11) |
| Propensity imputed HR | Ref | 0.95 (0.68, 1.33) |

* Adjusted age, sex, ethnicity, hypertension, diabetes obesity and baseline physiological observation score, CRP, neutrophil, chest x-ray abnormality, remdesivir and dexamethasone. . P values †= <0.001 # = <0.01 * = <0.05

## **Table 4c. Mortality rate and Cox proportional model hazard of death with Dexamethasone**

|  | No Dexamethasone | Dexamethasone |
| --- | --- | --- |
| Entire period of data collection - 28 day | n=2032 | n=1917 |
| Number of deaths | 384 | 301 |
| Unadjusted HR | Ref | 0.80 (0.69, 0.93) ^#^ |
| Fully adjusted HR | Ref | 1.01 (0.82, 1.25) |
| Propensity imputed HR | Ref | 0.97 (0.70, 1.35) |
| Entire period of data collection - 14 day | n=2032 | n=1917 |
| Number of deaths | 342 | 223 |
| Unadjusted HR | Ref | 0.66 (0.56, 0.79) ^†^ |
| Fully adjusted HR | Ref | 0.90 (0.71, 1.13) |
| Propensity imputed HR | Ref | 0.90 (0.62, 1.32) |
| Limited to Wave 2 - 28 day | n=745 | n=1869 |
| Number of deaths | 60 | 291 |
| Unadjusted HR | Ref | 1.99 (1.50, 2.62) ^†^ |
| Fully adjusted HR | Ref | 2.81 (1.82, 4.33) ^†^ |
| Propensity imputed HR | Ref | 1.05 (0.69, 1.59) |
| Limited to Wave 2 - 14 day | n=745 | n=1869 |
| Number of deaths | 52 | 213 |
| Unadjusted HR | Ref | 1.66 (1.22, 2.24) ^#^ |
| Fully adjusted HR | Ref | 2.17 (1.37, 3.45) ^#^ |
| Propensity imputed HR | Ref | 0.93 (0.59, 1.48) |

* Adjusted age, sex, ethnicity, hypertension, diabetes obesity and baseline physiological observation score, CRP, Neutrophil, chest x-ray abnormality, remdesivir and dexamethasone. P values ^†^= <0.001 ^#^ = <0.01 * = <0.05

## **Figure 4d. Kaplan Meier survival estimate, i) Remdesivir and ii) Dexamethasone**
